# SARS-CoV-2-Laden Respiratory Aerosol Deposition in the Lung Alveolar-Interstitial Region Is a Potential Risk Factor for Severe Disease: A Modeling Study

**DOI:** 10.3390/jpm11050431

**Published:** 2021-05-19

**Authors:** Sabine Hofer, Norbert Hofstätter, Albert Duschl, Martin Himly

**Affiliations:** Department of Biosciences, Paris Lodron University of Salzburg (PLUS), 5020 Salzburg, Austria; sabine.hofer@sbg.ac.at (S.H.); norbert.hofstaetter@sbg.ac.at (N.H.); albert.duschl@sbg.ac.at (A.D.)

**Keywords:** aerosol transmission, COVID-19, disease initiation, droplet nuclei, epidemiology, etiology, ground glass opacity, pathogenesis, pathophysiology

## Abstract

COVID-19, predominantly a mild disease, is associated with more severe clinical manifestation upon pulmonary involvement. Virion-laden aerosols and droplets target different anatomical sites for deposition. Compared to droplets, aerosols more readily advance into the peripheral lung. We performed in silico modeling to confirm the secondary pulmonary lobules as the primary site of disease initiation. By taking different anatomical aerosol origins into consideration and reflecting aerosols from exhalation maneuvers breathing and vocalization, the physicochemical properties of generated respiratory aerosol particles were defined upon conversion to droplet nuclei by evaporation at ambient air. To provide detailed, spatially-resolved information on particle deposition in the thoracic region of the lung, a top-down refinement approach was employed. Our study presents evidence for hot spots of aerosol deposition in lung generations beyond the terminal bronchiole, with a maximum in the secondary pulmonary lobules and a high preference to the lower lobes of both lungs. In vivo, initial chest CT anomalies, the ground glass opacities, resulting from partial alveolar filling and interstitial thickening in the secondary pulmonary lobules, are likewise localized in these lung generations, with the highest frequency in both lower lobes and in the early stage of disease. Hence, our results suggest a disease initiation right there upon inhalation of virion-laden respiratory aerosols, linking the aerosol transmission route to pathogenesis associated with higher disease burden and identifying aerosol transmission as a new independent risk factor for developing a pulmonary phase with a severe outcome.

## 1. Introduction

Virus shedding via virion-laden respiratory aerosol particles by contagious individuals has been confirmed for the common seasonal coronavirus, influenza virus and rhinoviruses [1,2]. Similarly, exposure to SARS-CoV-2-laden aerosols by sharing crowded indoor space can be expected as a substantial transmission risk with a seasonal pattern [3]. In the context of long-distance travel, this was demonstrated by a detailed analysis of 2334 index patients and 72,093 close contacts for high-speed train passengers in China [4]. The data revealed that the main determinants for transmission included co-exposure time and spatial distance. In occupational scenarios, the European Centre for Disease Prevention and Control reported that the vast majority (95%) of 447 investigated clusters occurred completely or predominantly indoors across all professional categories [5]. These reports are aligned with observations of cluster-forming events associated with recreational indoor activities and miscellaneous social gatherings [6,7]. It has also become increasingly acknowledged that aerosol transmission may play a significant, and not only a minor, role in confined spaces [8,9,10].

It has been demonstrated that SARS-CoV-2 is viable for hours in aerosols [11]. Experimental data on aerosol exhalation during different expiratory maneuvers describe size distribution, mechanisms of generation and sites of origin within the respiratory tract [12,13,14]. Due to the very specific particokinetic properties of aerosols derived from vocalization and breathing, the majority of these ambient air respiratory aerosol particles (ARAPs) resist gravitational settling and stay airborne for extended time [15,16]. Hence, virion-laden particles may contribute to short- and long-range airborne transmission by deposition and subsequent initiation of COVID-19 in the respiratory tract.

The etiology of COVID-19, however, has been predominantly linked with airborne droplet or contact transmission, with initial virus deposition and replication starting at the mucosa of the oral-nasal cavity followed by propagation through the upper respiratory tract epithelia to the alveolar-interstitial region with vulnerable cell types [17,18]. In cases with pulmonary involvement a stepwise migration via conducting airways towards the most distal regions of the lungs has been proposed [19,20]. This concept might need revision when considering transmission by immediately inhalable virion-laden aerosols with inherent potential for disease initiation in the peripheral lung. Outside the field of viral respiratory infections and with lung diseases caused by inhalable toxic particulate matter it is accepted that the site of initial deposition is also the site of initial adverse effects [21]. Such a reverse approach on disease propagation would have implications for pharmacological treatment considerations.

The hypothesis that ARAPs have the potential to initiate disease with pulmonary involvement, associated with a higher disease burden and characterized by pneumonia, dyspnea and reduced blood oxygen saturation [22], is supported by radiology findings on chest CTs. At the onset of the pandemic, classification of specific chest CT image anomalies has become key to support differential diagnosis of COVID-19-induced lung inflammation from pneumonia of other origin and the radiologist’s performance achieved sensitivity and specificity close to 90% [23,24]. Image classification by computerized deep learning methods, in particular convolutional neuronal networks, which rely on strong correlations between lesion areas in radiologic images and clinical indicators, achieve a specificity of >99% [25]. CT assessment includes the number, type, rate of anomaly development and particularly the distribution pattern in the lung. The predominant finding in chest CTs of patients (including presymptomatic patients) [26] was the peripheral distribution of ground-glass opacity (GGO) that occurred in the early stages of lung involvement [27]. This was described as a locally-confined, hazy increase in attenuation of secondary pulmonary lobules (units of three to five terminal bronchioles contained by fibrous septa) caused by partial filling and collapse of alveoli, interstitial thickening and increased capillary blood flow [28]. The key features of GGO that were observed with high frequency were bilateral involvement, posterior part or lower lobe predilection, prominent peripheral and sub-pleural distribution, absence of peri-bronchial localization [27]. Peripheral GGOs were observed in 86% (18/21) [29] and 80% (175/219) [30] of patients in two cohorts in China. In a longitudinal study of 90 cases where temporal changes in GGOs were investigated [31], the authors observed a gradual dissemination from unilateral to bilateral lung involvement with an increasing number of lesions and afflicted lobes. At the onset of symptoms, the abnormalities were predominantly sub-pleural with a peak at day 6 to 11. Another study of 121 cases that also mapped data on GGOs, demonstrated increased bilateral involvement with time (early-phase 28%, 10/36; late-phase 88%, 22/25) [32]. The highest frequency was observed in both lower lobes (65%, 79/121; 63%, 76/121).

**Figure 1:**
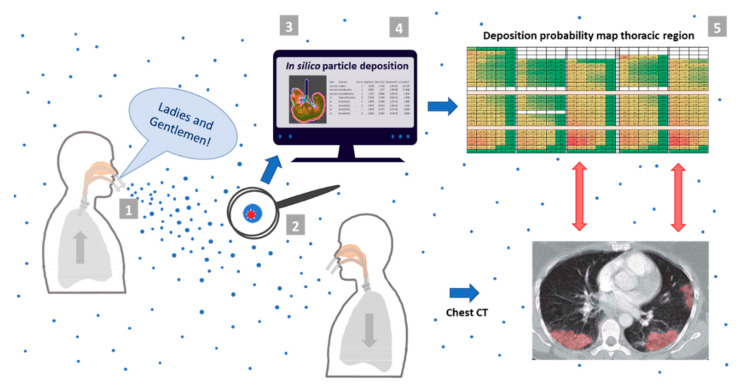
Schematic representation of the study concept and modeling. Virion-laden aerosol particles that are exhaled in the course of vocalization are immediately converted to ambient air respiratory aerosol particles (ARAPs) and remain airborne for hours. In silico modeling was used to determine whether these particles, if inhaled by exposed individuals, advance to the thoracic region and lead to preferred deposition in the distal regions of the lung. The spatial correlation of deposition hot spots with CT anomaly pattern (marked in red) would confirm disease initiation in the peripheral lung by these aerosols. For in silico simulation a five-step modeling and analysis procedure (indicated by grey boxes) was performed: step one—defining the physicochemical properties of exhaled respiratory aerosol particle upon generation; step two—establishing the physicochemical and particokinetic properties of these particles after conversion to ARAPs by evaporation to equilibrium with ambient air conditions; step three—establishing a detailed lung data model for ARAP deposition based on the Yeh/Schum 5-Lobe model [33]; step four—in silico simulation of ARAP deposition via Multiple Path Particle Dosimetry model [34,35] and International Commission on Radiological Protection model [36]; step five—refining resulting raw data by a top-down approach to provide detailed, spatially-resolved information on particle deposition in the thoracic region of the lung.

With mounting evidence and intensified debate on the role of aerosol transmission and involvement thereof in disease etiology and pathophysiology, it is compelling to combine in silico aerosol lung deposition modeling to supplement in vivo clinical observations and to generate a synergistic new view (Figure 1).

Literature research reveals that little is known about the potential of SARS-CoV-2-laden respiratory aerosols for direct inoculation and disease initiation in the human lung. Elucidation of the key events in the initiation of COVID-19 would be highly desirable, e.g., understanding the potential contribution of virion-laden aerosols to the tissue-delivered dose; and spatially-resolved distribution of their deposition onto lung epithelia of the bronchial, bronchiolar and alveolar region. Hence, to establish a spatially-resolved (detailed lung generation vs. ARAP size) deposition heat map, a five-step in silico modeling of virion-laden aerosols originating from vocalization combined with modeling of respiratory tract deposition was performed. Our study specifically focused on aerosols that originate from vocalization maneuvers, and were chosen because these aerosols are available for transmission via ambient air for hours and possibly generated by asymptomatic, but infected individuals. In addition, it has been proposed that, to a large extent, these aerosol size characteristics are not dependent on vocalization frequency and amplitude; thus, they are sex and age independent [37].

## 2. Materials and Methods

**Figure 2:**
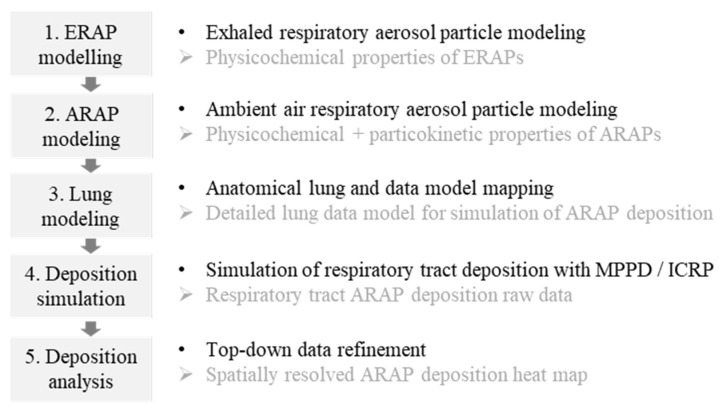
Overview of the five modeling and analysis steps. Bullet points: • activity; ➢ outcome. ERAP, exhaled respiratory aerosol particle; ARAP, ambient air respiratory aerosol particle; MPPD, Multiple Path Particle Dosimetry model; ICRP, International Commission on Radiological Protection model.

A stepwise modeling and analysis procedure was performed as depicted in Figure 2.

### 2.1. Respiratory Aerosol Particle Modeling

The respiratory aerosol particle (RAP) modeling is based on the aerosol size distribution studies of Morawska et al. [14] and Johnson et al. [12] for RAPs originating from human exhalation activities. Their results are in good agreement with reported particle size distributions from human activity [38]. From this comprehensive set of exhalation maneuvers that also included coughing, our investigation was limited to RAP data representing vocalization and breathing. To generate the data, the authors basically combined two experimental systems: an expiratory droplet investigation system (EDIS) that includes an aerodynamic particle sizer (APS) to measure from 0.5 µm to 20 µm; and a droplet deposition analysis (DDA) to measure particles larger than 20 µm. The measured particle size distribution was then analyzed by curve fitting. Five distinct particle populations, represented by five size distributions with corresponding mid-point diameters, were identified and assigned to distinct exhalation maneuvers and anatomical origins. These five mid-point diameters, hereafter referred to as mode 1 to mode 5, were used for the in silico particle deposition simulation in our study.

At the point of exhalation, RAPs are in evaporation equilibrium with the presumable breath cloud, i.e., 90% (+/− 7%) relative humidity (RH) and 28°C (+/− 1°) [12]. With exposure to ambient air, these exhaled respiratory aerosol particles (ERAPs) immediately begin to converge at a new evaporation equilibrium. For ERAPs at room temperature (RT), a RH <60%, and a particle size ≤20 µm, the transition to ambient air respiratory aerosol particles (ARAPs) is in the order of milliseconds to tenths of seconds [13,14]. In the literature, ARAPs ≤5 µm are frequently termed “droplet nuclei”, and RAPs beyond this size as “droplets”. Nevertheless, the concept of droplet nuclei formation is not limited to these size thresholds. According to Nicas et al. [39], and Holmgren et al. [40], irrespective of particle size, the evaporation shrinkage factor from ERAPs to ARAPs was experimentally determined at 0.5. Required for the calculation of particokinetic parameters, the physicochemical properties for ERAP and ARAP were established. Each particle was defined as spherically shaped object containing a single virion (diameter 0.12 µm, density 1.2 g/cm^3^) [41] in association with respiratory mucus (ERAP) or mucus remnants, when being evaporated to an equilibrium with ambient air (ARAP). The definition of one virion per particle is supported by the literature which, for example, reports 10^6^−10^11^ viral RNAs per milliliter in COVID-19 patients’ sputum [42]. If the highest concentration is aerosolized in our 1.6 µm ERAPs (0.8 µm ARAPs), this results in less than 1 virion per aerosol particle. Thus, given that the adsorption of an individual virion by an aerosol is a random event, most particles are free of virions and the remaining are loaded by just one virion. This observation was long before confirmed by Couch et al. [43] in the context of adenoviruses, where the vast majority had only one virion and this broadly concurs with a review of Poon et al. [44]. As shown in Table 1, the composition of the mucus associated with ERAPs is defined [45].

Due to the negligible contribution of the virion volume to the ERAP volume, for all particle sizes (1.6, 3.6, 7.0, 11.0, 145.0 µm), the density of the ERAPs was assumed to be equivalent to the mucus density of 1.041 g/cm^3^. The density calculation for ARAPs was determined with an evaporation shrinkage factor of 0.5 and elimination of water content with the following Equations (1)–(5):

d_ARAP_ = EF × d_ERAP_ (1)

V_ARAP_ = 1/6 × π × d^3^_ARAP_ (2)

m _evaporated H2O_ = ρ _H2O_ × (V_ERAP_ − V_ARAP_) (3)

m_ARAP_ = m_ERAP_ − m_evaporated H2O_ (4)

ρ_ARAP_ = m_ARAP_/V_ARAP_ (5)

d, diameter; V, volume; m, mass; ρ, density; EF, evaporation shrinkage factor

Table 2 provides the calculated characteristics of ARAPs used in our in silico deposition simulation and refers to the anatomical origin during exhalation activity.

### 2.2. Lung Modeling

**Figure 3:**
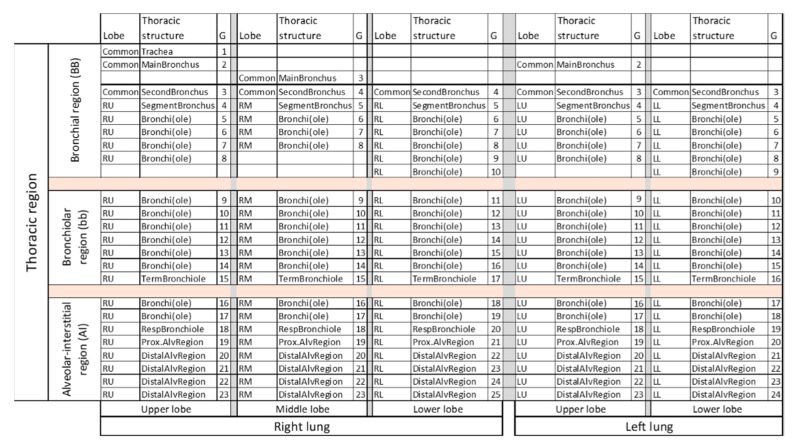
Anatomical structure of the thoracic region according to Yeh/Schum 5-Lobe model applied in the ARAP deposition simulation. Common structures are indicated on the far left. G, generation number; RU, right upper; RM, right middle; RL, right lower; LU, left upper; LL, left lower.

The simulation of ARAP deposition is based on the anatomical regions and airway generation model defined by International Commission on Radiological Protection (ICRP) [36] and the Yeh/Schum five-Lobe model [33]. In brief, the respiratory system is divided into four anatomical regions: the extrathoracic region, the bronchial region (BB), the bronchiolar region (bb) and the alveolar-interstitial region (AI) [47]. Together, BB, bb and AI constitute the thoracic region. The “Yeh/Schum 5-Lobe model” reflects an asymmetric human lung with five lobes and describes the airways within each lobe in a specific single-path manner. From this model, the human respiratory tract was ultimately compartmentalized into 110 regions; beginning with the common anatomical region of the trachea and expanding to the most distal alveolar region of each individual lobe of the lungs. Figure 3 depicts the anatomical segmentation of the thoracic region used in our simulation.

### 2.3. Deposition Simulation and Analysis

To generate the ARAP deposition heat map for each of the five particle modes, a series of calculations and models were performed as briefly described here. Firstly, an ARAP exposure dose by mass was defined and converted into an exposure dose by particle number. Secondly, Multiple Path Particle Dosimetry (MPPD) [34,35] was employed to simulate ARAP deposition per thoracic region. Thirdly, an ICRP-model was used to calculate the extrathoracic deposition and the in-/exhaled particle fractions. Finally, the combined data were used to calculate an ARAP deposition probability for a single inhaled ARAP to generate the final respiratory tract deposition heat map.

### 2.4. Simulation of Thoracic ARAP Deposition via in Silico Model MPPD

MPPD supports mathematical modeling of aerosol deposition in the human lung. Numerical solution of equations governing air flow, particokinetics and particle deposition is based on physical and physiological parameters of the lung and physicochemical determinants of aerosols under investigation. MPPD allows us to predict total, regional, lobar and local deposition in the lung. In our study we applied MPPD with the Yeh/Schum 5-Lobe lung morphometry model. It provides the advantage of a whole-lung model; while newer computational fluid dynamics models (CFD) are still regional, they are either limited to simulate the upper respiratory tract and airway deposition, hence, without the acinar lung generations, or deep lung simulation, they are missing aerosol deposition in the bronchial and bronchiolar region [48,49]. Yeh/Schum allows a good predictive accuracy for particle deposition in the deep lung, in good agreement and validated with in vivo experimental data or new CFD models like the 3D model of the deep lung, termed DLM [50]. Table 3 provides MPPD input parameters used:

For each distinct thoracic structure, the number of deposited particles per breath was extracted from the created MPPD detailed report (output field “Tot. Dep. Particle s (#)”). The sum of deposited particles over all thoracic structures per breath represents the overall number of deposited particles in the thoracic region per breath.

### 2.5. Calculation of Extrathoracic Deposition via the ICRP Model

Based on selected ARAP exposure concentration, particle mode and tidal volume used in the MPPD model, the particle number per breath was calculated for each specific ARAP mode. Extrathoracic deposited particle number per breath, and aerosol particle number not deposited, were calculated according to ICRP Equations (6) and (7) as follows:

IF = 1 − 0.5 × (1 − 1/(1 + 0.00076 × d^2.8^)) (6)

DF = IF × (0.0587 + 0.911/(1 + e^4.77+1.485×ln d^) + 0.943/(1 + e^0.503−2.58×ln d)^) (7)

TD = DF × TP

ETP = TD − TTD

NDP = TD − ETP − TTD

d, diameter; IF, inhalable fraction; DF, total deposited fraction; TD, total deposited particle number per breath; TP, particle number per breath; ETP, extrathoracic deposited particle number per breath; TTD, overall deposited particle number in the thoracic region per breath; NDP, not deposited particle number per breath.

### 2.6. Calculation of Deposition Probability for Thoracic Deposition Based on Total Deposited Particles

To establish the thoracic deposition probability, the number of deposited particles per breath for each specific ARAP mode was expressed in relation to the total deposited particle numbers per breath.

## 3. Results

**Figure 4:**
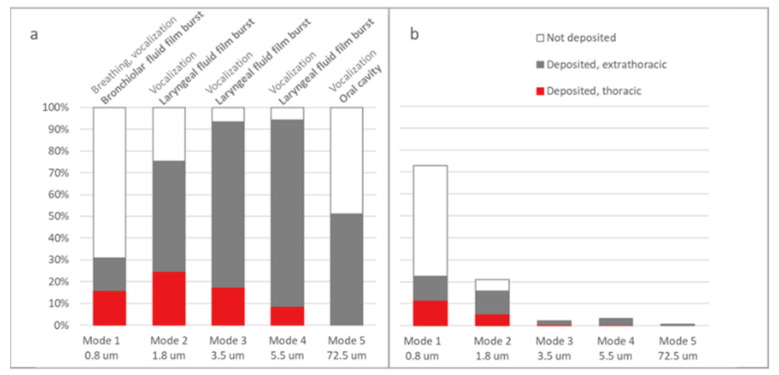
Respiratory tract deposition of the inhalable fraction of ARAPs; originating from exhalation activity “voiced counting”, a combined process of vocalization and breathing. These maneuvers resulted in an ARAP size distribution represented by five modes (0.8, 1.8, 3.5, 5.5, 72.5 µm) that originated from bronchiolar and laryngeal fluid film burst and the oral cavity. Depending on size, different fractions of ARAPs are deposited in the thoracic or extrathoracic region of the respiratory tract, or are not retained and exhaled. Deposition data are shown (**a**) unweighted and (**b**) weighted by relative abundance in the exhalation plume (mode 1–5: 72.8%, 21.0%, 2.2%, 3.4%, 0.6% [12,14]).

The ARAP respiratory tract deposition was analyzed at three different levels using a top-down approach. Five ARAP modes, exhaled during vocalization and breathing, represented by five size distribution modes that originated from bronchiolar and laryngeal region and the oral cavity were considered. Firstly, the overall deposition rate, that was segregated into extrathoracic and thoracic deposition fractions (Figure 4a), was investigated. Considerable thoracic deposition was observed for mode 1 (0.8 µm), mode 2 (1.8 µm) and mode 3 (3.5 µm). Mode 1 ARAPs were particularly notable with a high inhalation-exhalation rate of 69.1%. If the particles were retained, these also had the highest thoracic deposition probability of >50%. Across all ARAP modes, the ARAPs represented by mode 2 showed the highest absolute deposition probability for the thoracic region (24.8%). Mode 3 and mode 4 (5.5 µm) showed a predominant extrathoracic deposition; while mode 5 (72.5 µm) lacked any thoracic deposition. From all the particle modes generated through vocalization, mode 1 ARAPs captured the highest proportion and represented 72.8% of all ARAPs. This was followed by mode 2 (21.0%), and ARAP modes 3 to 5 contributed to 6.2% [12,14]. Weighting all modes against the relative proportion showed that only ARAP mode 1 and 2 contribute to thoracic deposition in vivo (Figure 4b).

**Figure 5:**
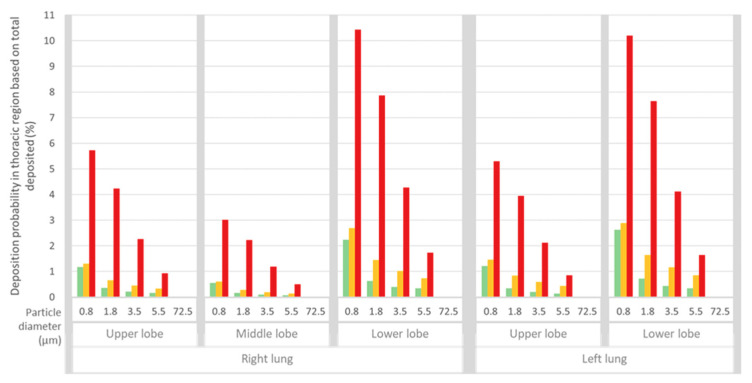
Deposition probability of ARAPs in the thoracic region, based on total deposited particle number (extrathoracic, thoracic). Deposition probability is shown for all five ARAP modes and is dissected further to the thoracic regions of the bronchial region (BB, green), bronchiolar region (bb, yellow) and alveolar-interstitial region (AI, red) for each lobe.

A more detailed approach was then performed to provide spatial distribution information on ARAP deposition. This was achieved by dissecting the information into lung lobe-specific data for the three thoracic respiratory tract regions BB, bb and AI. Irrespective of particle size, Figure 5 depicts a strikingly dominant deposition probability in the AI region of all lobes followed by bb, and then BB with the lowest deposition probability. The two lower lobes have a considerably higher burden than the middle right and the upper lobes; thus, indicating that the AI regions of the lower lobes are potential “hot spots” for ARAP deposition.

**Figure 6:**
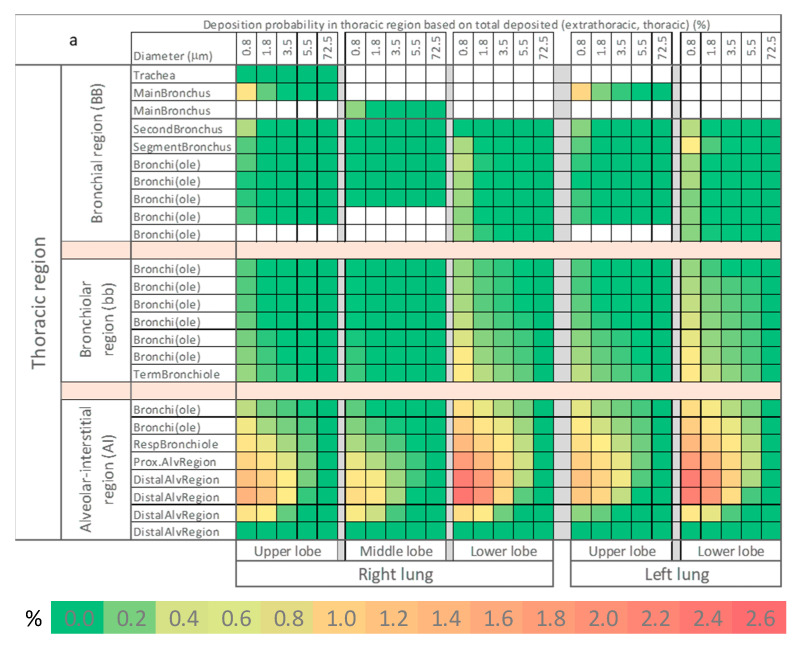

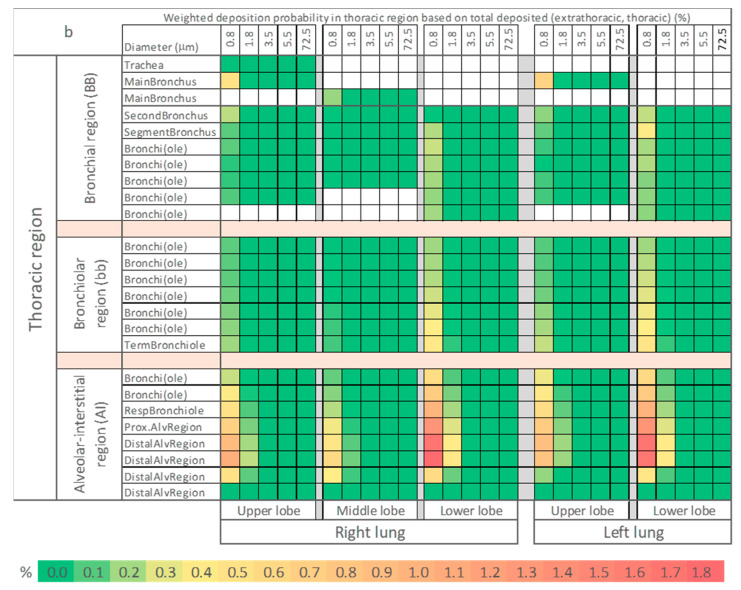
Heat map for the probability of ARAP deposition in the thoracic region based on total deposited particle number (extrathoracic, thoracic). Shown are all thoracic sub-structures and all ARAP modes. As lobes have different numbers of generations, the sub-structures were aligned. Common structures are indicated on the far left. Probability is color-coded according to legend. Probability of deposition is depicted (**a**) unweighted, and (**b**) weighted by relative abundance in the exhalation plume (mode 1–5: 72.8%, 21.0%, 2.2%, 3.4%, 0.6% [12,14]).

The third level in refining the analysis of ARAP deposition dissected the thoracic region into up to 25 lung generations for each lobe; beginning at common central structures and expanded into the most distal alveolar region. The two-dimensional (ARAP mode vs. lung lobe generation) deposition heat map (Figure 6a) immediately revealed deposition “hot spots” that begin at the proximal alveolar regions (PAR) of both lower lobes and intensify towards the distal alveolar regions until the penultimate generation. Taking into consideration the relative abundance of the different ARAP modes from vocalization, the weighted heat map (Figure 6b) again stressed the major contribution of ARAP mode 1 and the minor contributions of the other modes. Probability values and MPPD-derived raw data are available at http://doi.org/10.5281/zenodo.4736854, uploaded on 2 December 2020.

## 4. Discussion

Our modeling approach focuses on providing detailed data on deposition of virion-laden droplet nuclei in the thoracic region of the respiratory tract; thus, screening for anatomical sites with increased susceptibility for disease initiation. Data reveal that all ARAP modes that are associated with vocalization and originate from bronchiolar or laryngeal fluid film burst have the potential to be retained after inhalation. Conversely, the oral cavity-originating aerosols are not retained. Comparing the different sized ARAPs against relative abundance in the exhalation plume revealed that mode 1 and 2 ARAPs significantly contribute to a thoracic tissue-delivered dose; and ultimately, exclude all other modes. If mode 1 and 2 ARAPs alone are considered, then 43.9% of the deposited ARAPs appear in the thoracic region; thus, emphasizing the specific role of these ARAPs in the etiology of COVID-19 in vivo. To further elaborate the peculiarities of thoracic deposition, data were mapped to all five lung lobes. Calculating the deposition probability in the BB, bb and AI regions enabled differentiation of central from peripheral deposition. This analysis revealed the striking tendency of both lower lung lobes to be involved, and a preferential peripheral deposition in the AI region that exceeded central and BB deposition by up to three-fold. To date, this observed specific spatial pattern of ARAP deposition is aligned with the spatio-temporal distribution preferences of radiology chest CT anomalies in COVID-19 patients. Thus, at least in patients with pulmonary involvement, virion-laden ARAP inhalation seems to be linked to disease initiation. Finally, the chosen top-down modeling approach provided data refined from common central structures of all lobes to the most distal regions of 25 lung generations. The resulting deposition heat maps enabled recognition of deposition “hot spots” and the associated particle modes. The most prominent “hot spots” were observed in both lower lobes, beginning at the PAR and extending to the penultimate alveolar region. This was not different for our investigated aerosol upon rehydration to its size before evaporation started (ERAP), which would resemble the situation of regrowth after inhalation. The deposition probability values and heatmaps are available at http://doi.org/10.5281/zenodo.4736854, uploaded on 2 December 2020.

At this point, the correlation between spatially-resolved ARAP deposition and chest CT anomalies, predominantly peripheral COVID-19-associated GGOs in the absence of peribronchial GGOs, is evident and is in agreement with findings of GGOs as a phenomenon of secondary pulmonary lobules in early disease [28]. These specific sites in the peripheral lung, especially the PAR, are also a preferred area for alveolar and interstitial particle accumulation and inflammatory, infiltrative or tissue remodeling processes after exposure to low-toxicity, low-solubility inhalable aerosols [21]. Our results elucidate major differences in the tissue-delivered dose of virion-laden aerosols between the AI, bb, BB and extrathoracic regions. The study revealed a high probability of deposition of virion-laden aerosols at the alveolar epithelia in the AI region. Thus, we propose that these structures are particularly vulnerable to the initiation of adverse events after deposition of pathogenic near- and sub-micron-sized ARAPs. In the alveolar region, the protective barrier consists of a thin (average 200 nm) [51] fluid lining with an aqueous hypophase of low viscosity. At the cellular level, this means that each SARS-CoV-2 virion that is deposited by diffusion-driven mass transport can effectively and almost immediately interact with cellular receptors. A different scenario occurs when SARS-CoV-2 virion-laden aerosol particles are deposited on ciliated airway epithelia in the bb and BB regions. In healthy individuals, these areas are more effectively protected by a highly-viscous biphasic mucus layer with a thickness of approximately 20 µm [52]. A high clearance rate by the mucociliary escalator provides an outwardly-directed trajectory with a velocity exceeding 1 mm/min in the BB region. SARS-CoV-2 virions deposited on this mucus layer are dependent on non-directed mass transfer by diffusion towards the targeted epithelia. Physicochemical properties such as the viscosity and thickness of the mucus layer, however, markedly reduce the probability of timely epithelial contact by diffusional translocation; a prerequisite for subsequent disease initiation by viral entry. Due to the high mucus outward flow rate, a very low tissue-delivered dose can also be anticipated at the original site of deposition in the bb and BB regions. This is because the initially-deposited virion dose, that slowly penetrates the mucus layer via stochastic principles, would be distributed over a wide epithelial area resulting in a very high local dilution factor. Hence, implications in the early phases of COVID-19 pathogenesis at different epithelia should be expected. By taking into consideration the local dilution factor, the thickness and viscosity differences of the fluid linings in the AI and BB regions, the variation in disease initiation potential of a deposited virion dose could exceed a magnitude of 10^3^, which is also supported by Thomas et al. [38]. From the perspective of an immunologist, this would aid in explaining differences in disease severity. A low number of virus replicates, at early stage, in the presence of nevertheless sufficient viral antigen exposure in the BB region would initiate a standard immune response without excessive immune activation and with a low potential of advancing into the frequently-observed severe course of COVID-19.

A minimum infectious dose for SARS-CoV-2 is not established and is expected to be highly variable between different tissues, mainly depending on the availability of the viral-docking receptors and the major differences of protective barriers and clearance mechanisms. However, it is proposed for other viral respiratory infections that a single virus can serve for disease initiation [39]. More specifically, a recent study concerning the SARS-CoV-2 infectious dose in the oral-nasal-cavity proposed an infectious dose of 300 PFU [53]. Another key publication for SARS-CoV reported 43 PFU correlated to 10% infected individuals [54]. Overall, these studies propose that a low number of virions is sufficient for disease initiation. This assumption is practically underpinned by the 2020 Skagit Valley Chorale super-spreading incident, where a single COVID-19 carrier infected 52 out of 61 choral members, during a reported 2.5 h co-exposure [55].

Aside from the aforementioned aspects, the cellular regulation of viral docking receptors ACE2 and TMPRSS2 that moderate SARS-CoV-2 entry and, thus, the rate of viral replication in different lung epithelia, has to be considered. Noteworthily, ACE2 gene expression was found to be low in airway and alveolar epithelial cells in healthy individuals [17], but ACE2 gene was identified as interferon-stimulated in type II pneumocytes [56] upon viral challenge as part of a host-tissue protective mechanism, thereby particularly enhancing SARS-CoV-2 susceptibility and viral replication in the alveolar-interstitial region after an initial virus dose. This is well aligned with studies reporting ACE2 and TMPRSS2 co-expression in type II alveolar cells [57,58], but not in the upper airways [59]. Of note, new gene ontology data confirm ACE2 co-expression with immune response genes, in particular pro-inflammatory cytokines, chemokines and interferon γ-inducible protein 16 (IFI16) [60], an innate immune sensor regulating the interferon response to viral infections [61,62] by inflammasome activation. In this context, the particular susceptibility of type II pneumocytes is of major importance. These cells are progenitor cells for type I pneumocytes, and hence they are crucial for epithelial repair. Without restoration of alveolar tissue homeostasis COVID-19 can rapidly progress in disease severity with signs of diffuse alveolar damage and loss of functionality. This may in particular contribute to the increased fatality rate of the elderly age groups in SARS-CoV-2 acute lung injury, as demonstrated in a mouse model of alveolar epithelial type II cell senescence associated with impaired type II cell function in acute lung injury [63].

Limitations of our study are primarily due to the fact that the modeling presents an idealized version of actuality. In real life, virion-laden droplets would arrive with a possibly non-Gaussian size distribution. Although exhalation-generated aerosol particles are generally independent of body size, sex and age, the same cannot be said for inhalation; particularly when pre-existing lung conditions are taken into consideration with high variability in lung morphometry. These limitations have to be accepted applying a whole lung model operating at reasonable computational costs. Validation of in silico aerosol deposition models with in vivo experiments, by applying emerging techniques [64,65], would be desirable. For a general validation it would have to be done in healthy patients, which is probably a big hurdle for ethics review boards.

## 5. Conclusions

In conclusion, the stepwise modeling approach presented here showed that virion-laden ARAPs are primarily deposited in the secondary pulmonary lobules in lung generations beyond the terminal bronchiole. These regions are identical with the peripheral localization of GGOs observed in chest CTs; an early marker of COVID-19 infection. This agreement suggests that virion-laden ARAPs from vocalization maneuvers are significantly associated with COVID-19 pathology in the lung. Therefore, SARS-CoV-2 transmission via shared indoor aerosols appears to be an important route of infection in the peripheral lung and, thus, constitutes an independent risk factor for severe courses of disease. Our findings imply an alignment of public health care policies (i) to rebalance the risk of contact, large droplet and aerosol transmission, (ii) to address the particularity of indoor exposure and long-range transmission and (iii) to consider the substantial risk for more severe disease manifestations. Disease initiation in the alveolar-interstitial region, instead of the oral-nasal cavity with subsequent propagation towards the peripheral lung, implies optimization of treatment recommendations and disease monitoring. Both diagnosis of peripheral lung involvement early after COVID-19 confirmation by molecular tests, still in the asymptomatic stage, or early onset of pulmonary symptoms after confirmed or suspected exposure should be interpreted as a sign of disease initiation in the peripheral lung as primary anatomical site of infection. This pathogenesis imposes an increased likelihood for progression to severe outcome and, thus, increased public health care burden.

Linking our ARAP deposition results with recently provided respiratory viral emission estimation data [66] or with real world aerosol concentrations in indoor air would allow us to refine infection risk assessment and risk controlling strategies. Such combined models could be of great relevance for epidemiologists in the context of SARS-CoV-2 mutational variants of concern, such as 20B/501Y.V1, VOC 202012/01 (or B.1.1.7 lineage) with increased viral copy numbers [67] in the oral-nasal cavity. A higher fraction of virion-laden ARAPs with increased virion copy numbers increases the probability to pass the threshold for disease initiation, first of all, at the deposition hot spots identified here.

## Figures and Tables

**Table 1:** Mucus definition from composition data.

Component	Fraction Per Weight (%)	Mean Fraction Per Weight (%) ^(a)^	Density (g/cm^3^)
Protein (mucin)	2–5	3.5	1.35
Carbohydrate (glycan)	7.5–9	8.3	1.5
Lipid	1–2	1.5	0.985
Ions	1	1	1.409
H_2_O	80–90 ^(b)^	85.7	1
Mucus composition			1.041

^(a)^ mean fraction was used to calculate the density of ERAPs, ^(b)^ content decreases from airways in the lower to the upper respiratory tract [46].

**Table 2:** Ambient air respiratory aerosol particle characteristics used for in silico deposition simulation.

Mode	Diameter (µm)	Anatomical Origin [13]	Mass (g)	Volume (µm^3^)	Density (g/cm^3^)
Mode 1—breathing, vocalization	0.8	Bronchiolar fluid film burst	3.57 × 10^−13^	0.269	1.328
Mode 2—vocalization	1.8	Laryngeal fluid film burst	4.057 × 10^−12^	3.054	1.328
Mode 3—vocalization	3.5	Laryngeal fluid film burst	2.982 × 10^−11^	22.45	1.328
Mode 4—vocalization	5.5	Laryngeal fluid film burst	1.157 × 10^−10^	87.115	1.328
Mode 5—vocalization	72.5	Oral cavity	2.6507 × 10^−7^	199,532.040	1.328

**Table 3:** MPPD parameter settings for breathing scenario “light exercise”.

Input Section	Scenario	Parameter	Value Setting
Airway Morphometry	Aerosol	Model	Yeh/Schum 5-Lobe
Inhalant Properties	Constant Exposure	FRC	3300 mL
Exposure Condition		URT	50 mL
		Density	1.328 g/cm^3^
		Aspect Ratio	1.0 (=spherical)
		Diameter	0.8, 1.8, 3.5, 5.5, 72.5 µm ^(a)^
		Body Orientation	Upright
		Aerosol Concentration	0.5 mg/m^3 (b)^
		Breathing Frequency	15 per minute
		Tidal Volume	750 mL
		Inspiratory Fraction	0.5
		Pause Fraction	0
		Breathing Scenario	Oronasal-Normal Augmenter
		Deposition/Clearance	Deposition Only

^(a)^ One simulation per aerosol particle mode. ^(b)^ concentration used (only relevant for calculating deposition probabilities and not intended to reflect real world exposure). FRC, functional residual capacity; URT, upper respiratory tract.

## Data Availability

The data presented in this study or openly available in Zenodo at http://doi.org/10.5281/zenodo.4301641.
